# Soil Application of *Bacillus subtilis* Regulates Flavonoid and Alkaloids Biosynthesis in Mulberry Leaves

**DOI:** 10.3390/metabo14040180

**Published:** 2024-03-23

**Authors:** Yanfang Yu, Jinzhi Huang, Zhenhua Deng, Yawei Wang, Xinfeng Jiang, Junwen Wang

**Affiliations:** 1 Jiangxi Cash Crops Research Institute, Nanchang 330202, China; yuyf@jxjzs.freeqiye.com (Y.Y.); hjz4562023@163.com (J.H.); dzh126427@126.com (Z.D.); wangyawei588@126.com (Y.W.); jiangxinyue003@163.com (X.J.); 2 Jiangxi Provincial Key Laboratory of Plantation and High Valued Utilization of Specialty Fruit Tree and Tea, Nanchang 330202, China

**Keywords:** mulberry leaf, *Bacillus subtilis*, transcriptome, metabolomics, 1-deoxynojirimycin, flavonoid biosynthesis, alkaloids biosynthesis

## Abstract

Flavonoids and alkaloids are the major active ingredients in mulberry leaves that have outstanding medicinal value. *Bacillus subtilis* can effectively activate the plants defense response and regulate the plant secondary metabolism. In this study, we explored the effects of soil application of *B. subtilis* on the content of flavonoids and the most important alkaloids (1-deoxynojirimycin, DNJ) in mulberry leaves. Significant decreases in flavonoid content were observed in tender leaves and mature leaves after treatment with *B. subtilis*; at the same time, significant increases in DNJ content were observed in tender leaves. Based on widely targeted LC-MS/MS and high-throughput approaches, we screened out 904 differentially synthesized metabolites (DSMs) and 9715 differentially expressed genes (DEGs). KEGG analyses showed that these DSMs and DEGs were both significantly enriched in the biosynthesis of secondary metabolites, flavonoid synthesis and plant hormone signal transduction. Further correlation analysis of DEMs and DEGs showed that 40 key genes were involved in flavonoid biosynthesis, with 6 key genes involved in DNJ biosynthesis. The expression of CHS, CHI, F3H, F3′H, FLS, UGT and AOC significantly responded to *B. subtilis* soil application. This study broadens our understanding of the molecular mechanisms underlying the accumulation of flavonoids and alkaloids in mulberry leaves.

## 1. Introduction

Mulberry (*Morus* spp.) is an important cash crop that is widely cultivated in several countries [1,2]. Mulberry leaves have been utilized in Ayurveda and traditional Chinese medicine for treating various diseases, such as influenza, fever and diabetes [3,4]. In addition, they are often secondary sources of compounds used as medications, functional food and feed additives [5,6,7]. Flavonoids and alkaloids in mulberry leaves have received great attention due to their beneficial physiological effects, including antioxidant, hypoglycemic, anti-inflammatory, anti-hyperlipidemic activities, etc. [8,9,10]. 1-deoxynojirimycin (DNJ), recognized as the primary alkaloid in mulberry, has demonstrated promising therapeutic effects on diabetes and other related metabolic disorders [11]. Specially, DNJ is mainly found in mulberry and is very rare in other plants [11,12]. The biosynthesis of DNJ has garnered considerable interest recently. Previous data showed that leaf maturity significantly affects the DNJ content, exhibiting a distinct trend of young leaves having a higher DNJ content compared to mature leaves [13,14]. Our previous study demonstrated that DNJ content was significantly negatively correlated with the contents of phenolic compounds [15]. In addition, the contents of DNJ and phenolic compounds in mulberry leaves were affected by agricultural measures, such as different fertilization methods [16]. However, the synthesis and regulatory mechanisms of flavonoids and alkaloids in mulberry leaves have not been fully revealed.

*Bacillus subtilis* (BS) is a widely used type of plant-growth-promoting rhizobacteria (PGPR) that exhibits unique abilities in controlling plant diseases, inducing plant resistance, and promoting plant growth [17,18,19]. Previous studies have shown that BS could improve the absorption efficiency of nutrients by mulberry roots [20,21]. PGPR could produce and secrete different kinds of compounds that promote plant growth, including proteins, carbohydrates, signaling molecules, and some secondary metabolites, thereby leading to the change in metabolites and gene transcription levels of plants [22,23,24]. Researchers have found that the application of BS affects the production of flavanoids and alkaloids in plants [25,26,27,28]. Despite the potential significance of BS in affecting plant growth, there have been few studies exploring its effect on the secondary metabolism of mulberry leaves. To the best of our knowledge, the underlying molecular regulatory mechanisms of BS in the synthesis of plant secondary metabolites remain poorly understood.

In recent years, integrated metabolomics and transcriptomics analysis has become an efficient method to explore the synthesis mechanisms of plant secondary metabolites, such as phenolics compounds [29], flavonoids [30] anthocyanin [31] and terpenoid [32]. Integrated analyses were also used in the studies of flavonoid biosynthesis of mulberry leaves and have provided meaningful insights [33,34].

Thus, to further elucidate the mechanism underlying the effect of BS regulating the synthesis of flavonoids and DNJ in mulberry leaves, we conducted a pot experiment. The tender leaves (TL) and mature leaves (ML) of BS-treated mulberry and untreated mulberry were sampled for determining the contents of flavonoids and DNJ, followed by the integrated analysis of transcriptome and metabolomics. The gene–metabolite regulatory network was drawn. The related genes and metabolics of the flavonoid synthesis pathway and alkaloids biosynthesis pathway were analyzed. This study would provide critical guidance for the practical application of BS in medicinal plant cultivation. The results also contribute to understanding the synthesis pathway and regulatory mechanisms of flavonoids and alkaloids.

## 2. Materials and Methods

### 2.1. Plant Materials and Sample Preparation

One-year-old ‘Yuesang 11′ (*Morus atropurpurea* Roxb.) seedlings were planted in pots (inner diameter × height: 32 × 29 cm) in Nanchang, Jiangxi, China, on 26 February 2022. *B. subtilis* agent with an effective viable bacterial count of 2 × 10^10^ CFU/g was purchased from Henan Wobao Biotechnology Co., Ltd.(Hebi, China). *B. subtilis* suspension was prepared by mixing the agent with water according to the manufacturer’s manual. The pots were positioned within a greenhouse characterized by a transparent roof and open surroundings. Approximately 20 kg of soil, composed of yellow soil, peat and perlite at a ratio of 2:1:1, was put in a plastic pot. The pH value of the experimental soil was measured to be 6.75. The organic matter, total nitrogen, available potassium and available phosphorus contents were 29.34 g/kg, 2.04 g/kg, 214.00 mg/kg and 78.54 mg/g, respectively. Sixty mulberry trees with consistent growth were randomly divide into two groups, namely BS-treated and CK. Based on the preliminary experimental results, after five and seven months of regular cultivation, the soil of the BS-treated group was irrigated with 500 mL of *B. subtilis* suspension (2 × 10^7^ CFU/mL) per pot once. CK was irrigated with 500 mL distilled water. Then, after four weeks of the second treatment, the tender leaves (TL) and mature leaves (ML) of each mulberry tree were collected. The leaves were classified based on branch positions: the tender leaves were at the first to second position and the mature leaves at the fifth to sixth position from the branch apex [13]. In other words, there were four groups: CK-TL, CK-ML, Treat-TL and Treat-ML. We randomly selected 10 mulberry trees as the source of a biological duplicate sample. A total of three biological replicates were conducted for each group. About 5 g of fresh leaves of each sample was rapidly placed into a 50 mL nuclease-free centrifuge tube, promptly immersed in liquid nitrogen, and subsequently stored in a −80 °C freezer until the metabolome and transcriptome analyses were ready to be conducted. The other portion of the materials was used for determining the contents of total flavonoids and DNJ using the method described in our previous study [15].

### 2.2. Metabolomic Analysis

The samples were dried using a vacuum freeze-dryer (Scientz-100F, Scientz, Ningbo, China) and crushed for 1.5 min by a mixer mill (MM 400, Retsch Technology, Haan, Germany). For extraction, 100 mg of the powder was dissolved in 1.2 mL of 70% methanol solution, followed by vortexing for 30 s every 30 min for 6 times, and kept at 4 °C overnight. After centrifugation at 12,000 rpm for 10 min, the extracts were filtrated through a 0.22 nylon membrane (SCAA-104, ANPEL, Shanghai, China) before UPLC-MS/MS analysis.

The widely targeted metabolome analysis was performed using the LC-ESI-MS/MS system (UPLC, SHIMADZU Nexera X2, Kyoto, Japan; MS, 4500 Q TRAP, Applied Biosystems, Foster City, CA, USA) equipped with an ESI Turbo Ion-Spray interface. The chromatographic separations were meticulously performed using an Agilent SB-C18 column (1.8 µm, 2.1 mm × 100 mm). The column oven was set to 40 °C. The mobile phases were 0.1% formic acid water (phase A) and acetonitrile with 0.1% formic acid (phase B). The injection volume was 4 μL. The gradient elution system was operated at a consistent flow rate of 0.35 mL min^−1^. The gradient profile was precisely programmed as follows: starting conditions of 5% B, gradually increasing to 95% B within 9 min, and 95% B kept for 1 min, then adjusted back to 5% B within 1.1 min and kept for 2.9 min to re-equilibrate the column. Linear ion trap (LIT) and triple quadrupole (QQQ) scans were operated in both positive- and negative-ion modes. These scans were meticulously controlled using the Analyst 1.6.3 software (AB Sciex). The key source operation parameters were as follows: The ion source temperature was 550 °C. The ion spray voltage (IS) was 5500 V (positive ion mode)/−4500 V (negative ion mode). Ion source gas I (GSI), gas II (GSII), and curtain gas (CUR) were set at 50, 60, and 25.0 psi, respectively. Instrument tuning and mass calibration were meticulously performed using polypropylene glycol solutions at concentrations of 10 μmol/L for the QQQ mode and 100 μmol/L for the LIT mode. For the QQQ scans, multiple reaction monitoring (MRM) experiments were conducted, with the collision gas (nitrogen) set to a medium flow rate. The Declustering Potential (DP) and Collision Energy (CE) for individual MRM transitions were optimized to maximize sensitivity and selectivity. Additionally, a specific set of MRM transitions was monitored for each elution period, tailored to the metabolites present within that period.

A log2 transformation was applied to normalize the metabolite data. All metabolite data from the samples were subjected to principal component analysis (PCA) via R 3.5.1 software (www.r-project.org/, accessed on 11 November 2023), and unit variance (UV) scaling was used to preprocess the data. Orthogonal partial least squares discriminant analysis (OPLS-DA) was performed to further investigate the differences between groups. The *p*-value was set to 0.05, and a fold change threshold of 2.0 was employed to identify metabolites with significant changes. Venn diagrams were used to depict the number of differentially synthesized metabolites (DSMs). Hierarchical clustering heatmap of alkaloids and flavonoids in different groups were performed using the Metware Cloud (https://cloud.metware.cn, accessed on 24 January 2024). The Kyoto Encyclopedia of Genes and Genomes (KEGG) database with a *p*-value < 0.01 was used to study DSMs in the BS-treated mulberry leaves compared to CK (CK-MLvsTreat-ML, CK-TLvsTreat-TL). All data were graphed using GraphPad Prism v6.01 (GraphPad Software Inc., La Jolla, CA, USA).

### 2.3. Transcriptomic Analysis

The RNAprep Pure Plant kit (Tiangen Biotech., Beijng, China) was utilized for the isolation of total RNA from the samples. Subsequently, Illumina RNA-Seq library preparation was carried out by Metware Biotechnology Co., Ltd., located in Wuhan, China. To ensure the integrity and purity of the RNA, it was subjected to quality checks using a NanoPhotometer spectrophotometer (IMPLEN, Calabasas, CA, USA), a Qubit 2.0 Fluorometer (Life Technologies, Carlsbad, CA, USA), and an Agilent Bioanalyzer 2100 system (Agilent Technologies, Santa Clara, CA, USA). To enrich poly (A) mRNA, magnetic beads coated with oligo (dT) were employed. The mRNA underwent random fragmentation, followed by the synthesis of the first-strand cDNA using the M-MuLV reverse-transcriptase system. Subsequently, the RNA strands were degraded by RNase H, enabling the synthesis of the second-strand cDNAs by DNA polymerase. The resulting double-stranded cDNAs were ligated to sequencing adapters. AMPure XP beads were employed to select cDNAs with a size of approximately 200 base pairs. The cDNA libraries were prepared for sequencing on the Illumina Novaseq6000 platform after amplification and purification.

To ensure the integrity and reliability of the data obtained, adapters were excised from the sequences to eliminate any potential artefacts introduced during library preparation. Subsequently, reads with a substantial proportion of uncertain bases, specifically those containing ≥ 5 ambiguous bases or exhibiting over 50% of their bases with a Phred quality score ≤ 20 (indicating low-quality data), were excluded from further analysis. Then, the GC-contents of the cleaned reads were computed. The Q20 and Q30 values were generated using FastQC. These values represent the percentage of bases in the reads with a Phred quality score ≥20 and ≥30, respectively. Subsequently, the refined reads were aligned to the reference genome of the hickory species utilizing HISAT2. The mapping process involved aligning the cleaned reads to the reference genome using default parameters. The annotation of unigenes was achieved through a comprehensive alignment process with multiple databases, including Gene Ontology (GO), SwissProt, the non-redundant (Nr), eukaryotic orthologous groups (KOG) of proteins, and KEGG databases using Diamond. The quantification of gene expression levels was achieved through the FPKM (fragments per kilobase of transcript per million fragments mapped) approach. Orthogonal Partial Least Square Discriminant Analysis (OPLS-DA) was employed to distinguish the overall differences. Differentially expressed genes (DEGs) were identified according to the thresholds of corrected *p*-value < 0.05 and |log2FoldChange| > 2. KEGG enrichment analysis was performed to identify enriched biological pathways. Additionally, Gene Ontology (GO) analysis was conducted to annotate and categorize the DEGs based on their biological functions.

### 2.4. Integrative Analysis of Metabolomics and Transcriptome

A comprehensive mapping of all differentially expressed genes (DEGs) and differentially abundant metabolites (DSMs) was conducted onto the KEGG pathway database. Two-way orthogonal Partial Least Square with Discriminant Analysis (O2PLS) analysis was conducted to select the important DEGs and DSMs. Metabolites and DEGs involved in flavonoids and DNJ biosynthesis were selected for further analysis. Pearson’s correlation test was performed to investigate the correlations between the selected metabolites and their corresponding genes.

### 2.5. RT-PCR Analysis

Nine DEGs highly correlated with flavonoid and alkaloids synthesis were verified via the qRT-PCR method. Next, 1 µg of each qualified RNA sample was used for the reverse-transcription reaction. The RNA samples were reverse-transcribed to first-strand cDNA with a TransScript^®^ Uni All-in-One First-Strand cDNA Synthesis SuperMix for qPCR kit (TransGen Biotech., Beijing, China) according to the manufacturer’s instructions. The primers utilized in this study are comprehensively listed in Table S1. The qRT-PCR method was conducted with a Magic SYBR Mixture qPCR kit (Cwbio Bio., Beijing, China). The reaction system was as follows: 1 µL of cDNA, 10 µL of 2 × PerfectStart Green qPCR SuperMix, 0.4 µL of forward primer (10 µM), 0.4 µL of reverse primer (10 µM), and 8.2 µL of nuclease-free water. The qRT-PCR was performed on an Archimed-X4 fluorescence quantitative PCR instrument (ROCGENE, Beijing, China). Relative transcript levels were determined using the 2−ΔΔCt method, with β-Actin gene serving as the reference gene. The experiments were replicated both biologically and technically, with a total of three replicates performed for each sample. The data were graphed using OriginPro 9.1 software (OriginLab Corp., Northampton, MA, USA).

## 3. Results

### 3.1. The Changes in Flavonoid and DNJ Contents in Mulberry Leaves

**Figure 1:**
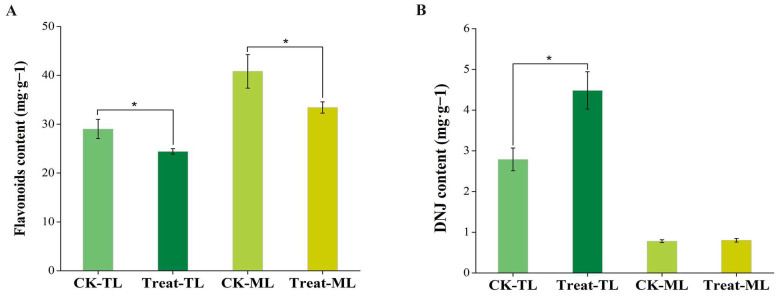
Total flavonoid content (**A**) and 1-deoxynojirimycin content (**B**) of mulberry leaves. The asterisks above the horizontal lines represent significant differences between the groups (*p* < 0.05). CK-TL, tender leaves of control group; CK-ML, mature leaves of control group; Treat-TL, tender leaves of the *B. subtilis*-treated group; Treat-ML, mature leaves of the *B. subtilis*-treated group.

As shown in Figure 1, the DNJ content of BS-treated tender leaves (Treat-TL) increased significantly (*p* < 0.05) compared with the control tender leaves (CK-TL), while the flavonoid levels decreased significantly (*p* < 0.05). Similarly, the total flavonoid content of BS-treated mature leaves (Treat-ML) was lower than that of the control mature leaves (CK-ML), but no significant difference was observed in the DNJ content between Treat-ML and CK-ML.

### 3.2. Diverse Metabolites Enrichment in Mulberry Leaves after B. subtilis Treatment

**Figure 2:**
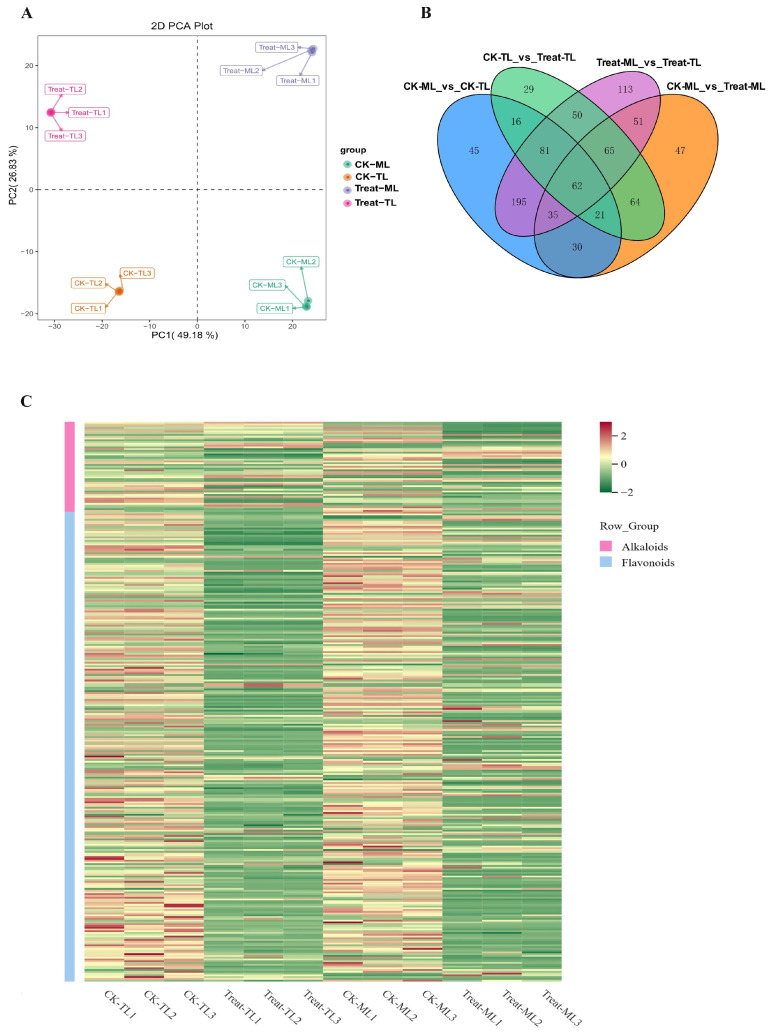
(**A**) Plots of the principal component analysis (PCA) of the differentially synthesized metabolites (DSMs). (**B**) Venn diagram for all DAMs in CK-TL, CK-ML, Treat-TL and Treat-ML. Each circle represents a comparison group. The number of overlapping parts represents the number of common differential metabolites between the comparison groups, and the number without overlapping parts represents the number of specific differential metabolites in the comparison group.(**C**) Hierarchical clustering heatmap of alkaloids and flavonoids in CK-TL, CK-ML, Treat-TL and Treat-ML.

Through the analysis of the widely targeted metabolome of all samples, a total of 1292 metabolites were obtained (Table S2), which were distinctively clustered into four groups according to the PCA analysis (Figure 2A). The first principal component and the second principal component can explain 49.18% and 26.83% of the total variance. Moreover, 904 differentially synthesized metabolites (DSMs) were found, which were mainly classified into alkaloids, flavonoids, amino acids and derivatives, lignans and coumarins, nucleotides and derivatives, lipids, organic acids and others (Table S3). Next, 62 common differential metabolites across the 4 groups were obtained (Figure 2B). Using a fold-change threshold of >2.0, we identified 388 and 375 up-regulated metabolites in CK-MLvsTreat-ML and CK-TLvsTreat-TL, respectively. There were obvious differences in flavonoid and alkaloid contents between groups, as shown in the heat map (Figure 2C). Notably, almost all the flavonoids were significantly down in CK-MLvsTreat-ML and CK-TLvsTreat-TL (Figure 2C, Table S3).

**Figure 3:**
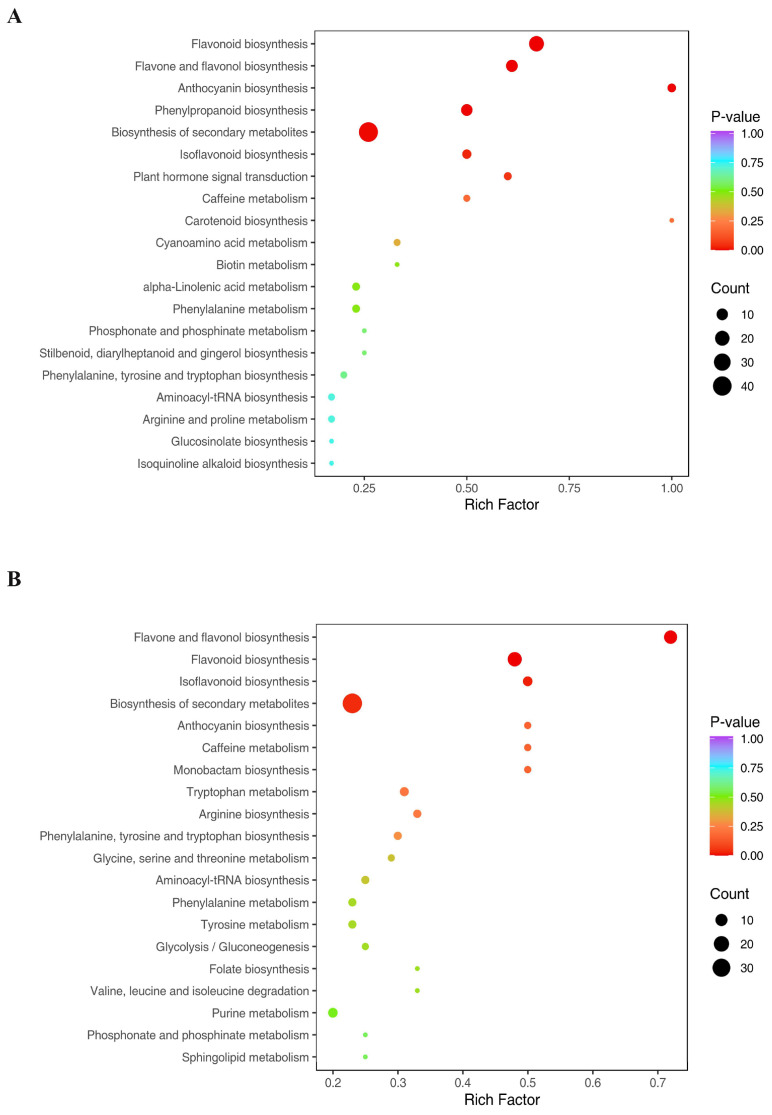
KEGG enrichment analysis of differentially synthesized metabolites (DSMs) in CK-TLvsTreat-TL (**A**) and CK-MLvsTreat-ML (**B**). The sizes and colors of the dots represent the number of metabolites and the significance (*p*-value), respectively.

The metabolites were then assigned to KEGG pathways. The main KEGG pathway enriched by DSMs in all the samples was found to be the biosynthesis of secondary metabolites. The top ranked DSMs in CK-MLvsTreat-ML (Figure 3A) and CK-TLvsTreat-TL (Figure 3B) were associated with the biosynthesis of secondary metabolites, flavonoid synthesis, flavone and flavonol biosynthesis.

### 3.3. Transcriptome Changes in Mulberry Leaves after B. subtilis Treatment

**Figure 4:**
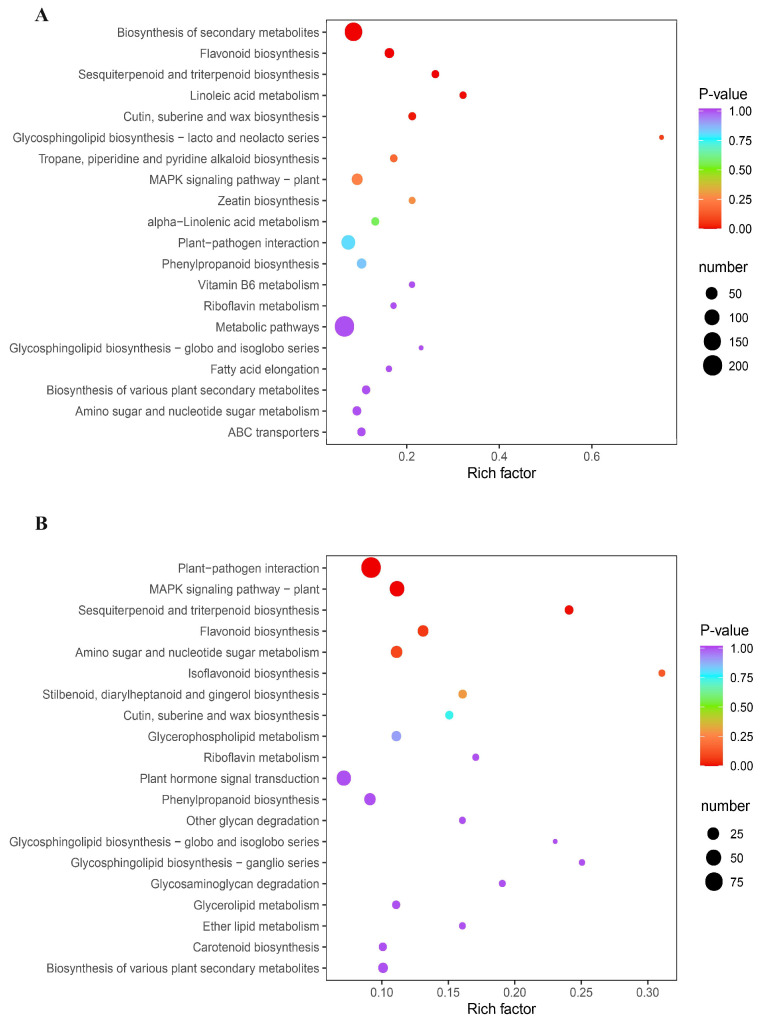
KEGG enrichment analysis of the differentially expressed genes in CK-TLvsTreat-TL (**A**) and CK-MLvsTreat-ML (**B**). The sizes and colors of the dots represent the number of genes and the significance (*p*-value), respectively.

A total of 83.49 GB clean reads were obtained after filtering out the low-quality reads. The amount of effective data obtained from each sample varied between 6.42 and 7.47 GB. The quality score (Q30) exceeded 91% for all samples. Furthermore, the average guanine–cytosine (GC) content was 45.60%, indicating high-quality transcriptome sequencing data. Upon functional annotation, a total of 25,570 genes were identified (Table S4). Next, 9715 differentially expressed genes (DEGs) were found; more specifically, 1307 (666 down- and 641 up-regulated) and 1432 (674 down- and 758 up-regulated) DEGs were screened out in the pairwise comparisons of CK-MLvsTreat-ML and CK-TLvsTreat-TL, respectively (Table S5). KEGG analysis revealed the significant enrichment of DEGs in various biological pathways, including metabolic pathways, the biosynthesis of secondary metabolites, plant hormone signal transduction and plant–pathogen interaction in CK-TLvsTreat-TL (Figure 4A) and CK-MLvsTreat-ML (Figure 4B).

**Figure 5:**
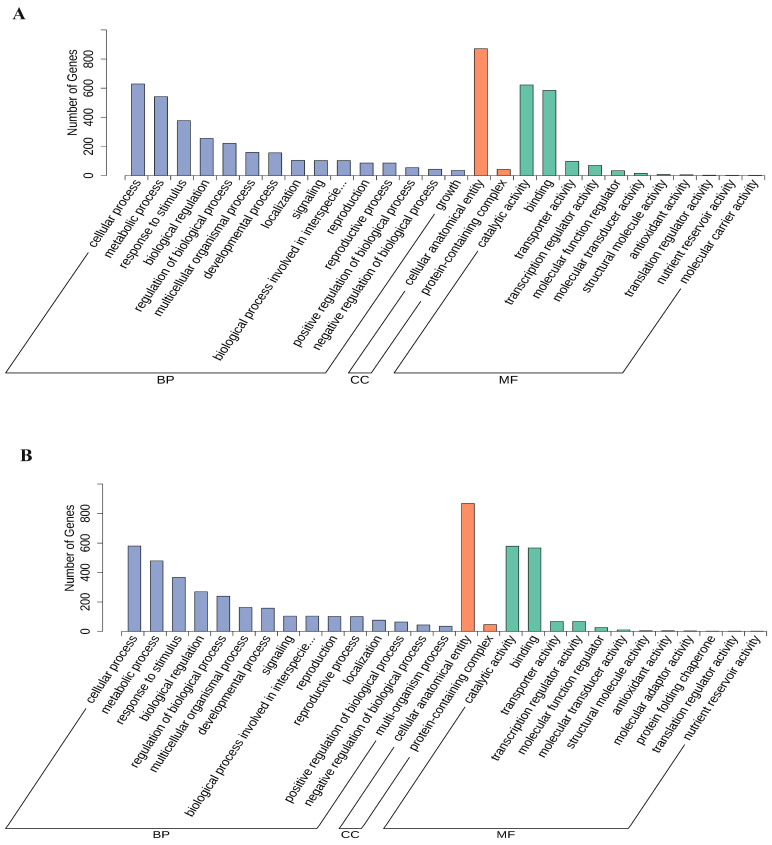
GO function classification of the differentially expressed genes in CK-TLvsTreat-TL (**A**) and CK-MLvsTreat-ML (**B**). BP, biological process; CC, cellular component; MF, molecular function.

The GO annotations of all DEGs were organized into three categories: biological process (BP), cellular component (CC) and molecular function (MF). In the BP category, most of the DEGs of CK-TLvsTreat-TL and CK-MLvsTreat-ML were enriched in the cellular process, metabolic process and response to stimulus. In the CC category, almost all of the DEGs were enriched in cellular anatomical entity and protein-containing complex. In the MF category, most of the DEGs were enriched in catalytic activity and binding terms (Figure 5).

### 3.4. Correlation between DSMs and DEGs

The combined KEGG analysis of DSMs and DEGs was further analyzed. The results showed that both the DSMs and DEGs were enriched in the metabolic pathways, biosynthesis of secondary metabolites, flavonoid synthesis and plant hormone signal transduction, whether CK-MLvsTreat-ML or CK-TLvsTreat-TL (Figure S1). The top DSMs (Table S6) and DEGs (Table S7) were screened out via O2PLS analysis. Almost all of the top metabolites were flavonoids. The top 10 DEGs, including LOC21396121 (gibberellin-44 dioxygenase), LOC21386582 (multidrug resistance protein), LOC21409922 (heat shock transcription factor), LOC21395522 (peptide/histidine transporter) and novel2146 (serine/threonine-protein kinase PBS1), were related to plant–pathogen interaction and the biosynthesis of secondary metabolites.

**Figure 6:**
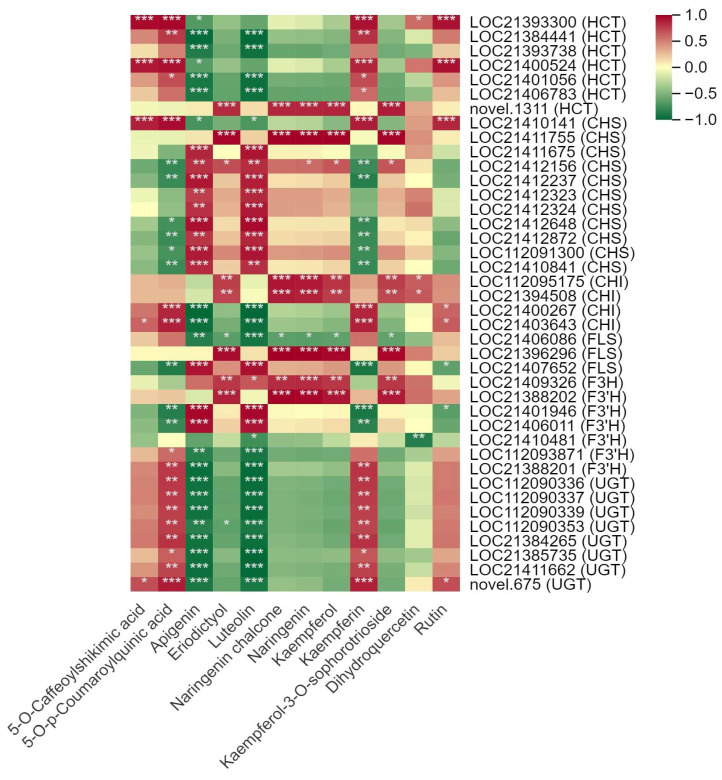
Correlation between the main differentially synthesized metabolites (DSMs) and differentially expressed genes (DEGs) in the flavonoid biosynthesis pathway in mulberry leaves. One asterisk (*) represents *p* ≤ 0.05, two asterisks (**) represent *p* ≤ 0.01, and three asterisks (***) represent *p* ≤ 0.001.

Pearson’s correlation analysis demonstrated a significant association between the DSMs and DEGs involved in the biosynthesis of flavonoids and alkaloids biosynthesis (Table S8). In total, 12 DSMs were screened out in the biosynthesis of quercetin glycosides and kaempferol glycosides, which were the predominant flavonoids of mulberry leaves. A total of 40 DEGs, mainly encode hydroxycinnamoyl-coenzyme A shikimate/quinate hydroxycinnamoyl transferase (HCT), chalcone synthase (CHS), chalcone isomerase (CHI), flavonol synthase (FLS), flavanone 3-hydroxylase (F3H), flavonoid 3′-monooxygenase (F3′H) and UDP-glycosyltransferase (UGT), were related to the 12 DSMs (Figure 6). 5-O-caffeoylshikimic acid, 5-O-p-coumaroylquinic acid, kaempferin and rutin were positively correlated with 24 DEGs (6 HCT, 1 CHS, 4 CHI, 2 FLS, 3 F3′H, 8 UGT). In contrast, apigenin, eriodictyol, luteolin, naringenin chalcone, naringenin, kaempferol and kaempferol-3-O-sophorotrioside were generally negatively associated with the above 24 DEGs (except LOC112095175, LOC21394508, LOC21407652, LOC21388202) but positively correlated with the other 16 DEGs (1 HCT, 10 CHS, 1 FLS, 1 F3H, 3 F3′H).

**Figure 7:**
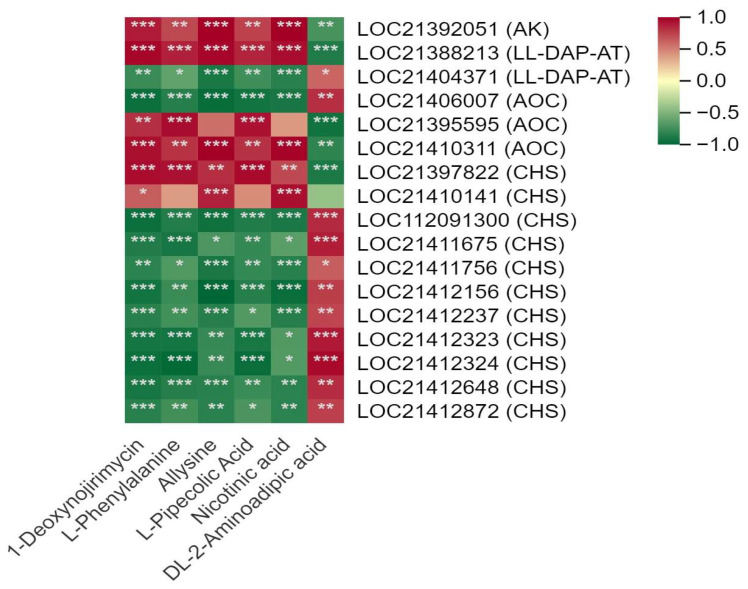
Correlation between the main differentially synthesized metabolites (DSMs) and differentially expressed genes (DEGs) located in the 1-deoxynojirimycin biosynthesis pathway in mulberry leaves. One asterisk (*) represents *p* ≤ 0.05, two asterisks (**) represent *p* ≤ 0.01, and three asterisks (***) represent *p* ≤ 0.001.

A total of 14 DEGs, mainly encoding CHS and primary amine oxidase (AOC), were involved in the alkaloid biosynthesis pathway (Figure 7, Table S8). Among them, two AOC transcripts (LOC21395595, LOC21410311) and two CHS transcripts (LOC21397822, LOC21410141) were positively correlated with DNJ. The other eight CHS transcripts were generally negatively with DNJ and L-phenylalanine. In addition, aspartate kinase (AK) and LL-diaminopimelate aminotransferase (LL-DAP-AT) located in the lysine biosynthesis pathway were observed to be significantly correlated with DNJ and L-phenylalanine.

### 3.5. Analysis of the DEGs in the Flavonoid and Alkaloids Synthesis Pathways

**Figure 8:**
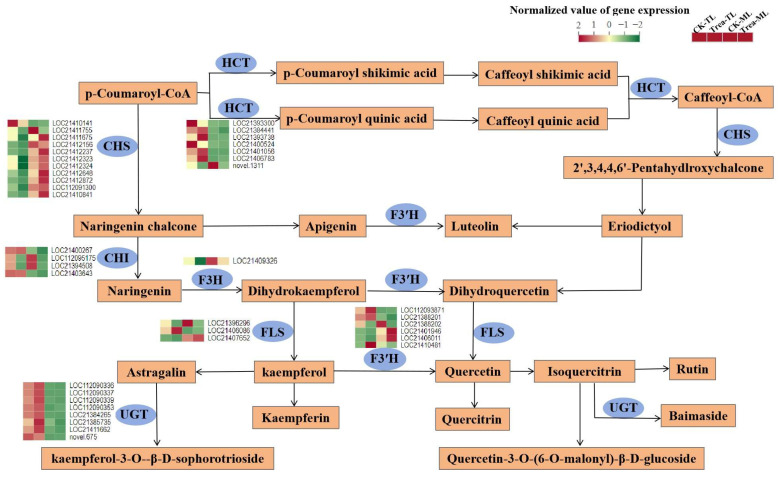
Putative flavonoid biosynthesis pathway and transcription levels of related enzyme genes in mulberry leaves. HCT, hydroxycinnamoyl-coenzyme A shikimate/quinate hydroxycinnamoyl transferase; CHS, chalcone synthase; CHI, chalcone isomerase; F3H, naringenin 3-dioxygenase; FLS, flavonol synthase; F3′H, flavonoid 3′-monooxygenase; UGT, UDP-glycosyltransferase.

Among the DEGs located in flavonoid pathway (Figure 8, Table S9), almost all of the CHS expression levels were significantly lower in tender leaves (CK-TL, Treat-TL) than those in mature leaves (CK-ML, Treat-ML). In contrast, most of the HCT and UGT expression levels were significantly lower in mature leaves than those in tender leaves. Compared to CK-TL, a total of 10 DEG (7 CHS, 1 F3′H, 1 FLS, and 1 HCT) were significantly down-regulated in Treat-TL. Meanwhile, a total of 10 DEG (1 CHS, 3 CHI, 1 FLS, 2 F3′H, 2 HCT and 1 UGT) were significantly down-regulated in Treat-ML than CK-TL.

**Figure 9:**
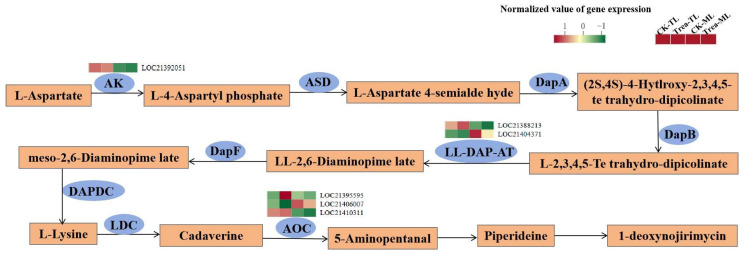
Putative 1-deoxynojirimycin biosynthesis pathway and transcription levels of related enzyme genes in mulberry leaves. AK, aspartate kinase; ASD, aspartate–semialdehyde dehydrogenase; DapA, 4-hydroxy-tetrahydrodipicolinate synthase; DapB, 4-hydroxy-tetrahydrodipicolinate reductase; LL-DAP-AT, LL-diaminopimelate aminotransferase; DapF, diaminopimelate epimerase; DAPDC, diaminopimelate decarboxylase; LDC, L-lysine decarboxylase; AOC, primary amine oxidase.

Among the DEGs located in DNJ biosynthesis pathway (Figure 9, Table S9), LOC21392051 (AK), LOC21388213 (LL-DAP-AT) and LOC21410311 (AOC) expression levels were significantly higher in tender leaves than those in mature leaves, but LOC21404371 (LL-DAP-AT) and LOC21406007 (AOC) expression levels were opposite. Notably, LOC21395595 (AOC) was significantly up-regulated in Treat-TL compared to CK-TL.

### 3.6. qRT-PCR Validation of Gene Expression

**Figure 10:**
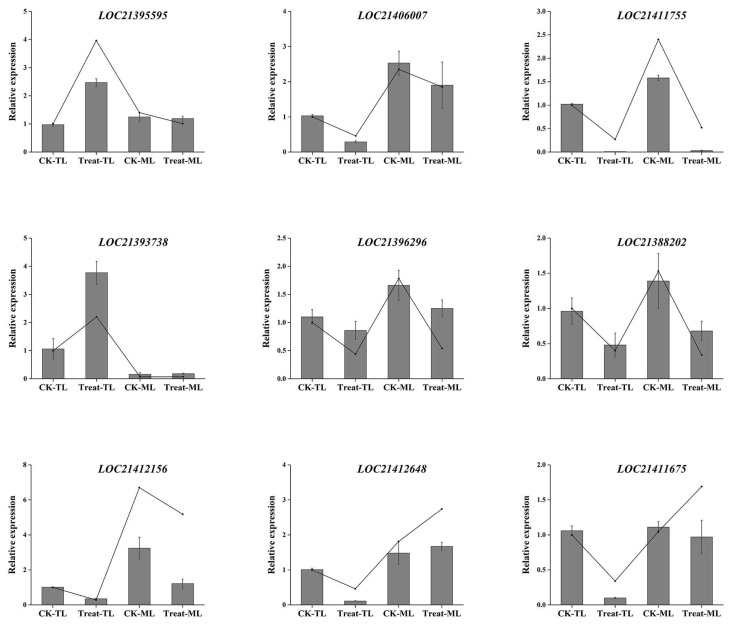
Relative transcription levels and expression levels of selected genes in mulberry leaves. The lines represent fragments per kilobase per million (FPKM) of the genes. The bar charts represent the relative expression levels validated via qRT-PCR.

We analyzed the expression levels of nine DEGs highly correlated with flavonoid and alkaloids synthesis via the qRT-PCR method. The results showed that the expression levels of LOC21395595 (AOC) and LOC21393738 (HCT) in Treat-TL were significantly higher than that in CK-TL (Figure 10). The expression levels of LOC21406007 (AOC), LOC21411755 (CHS), LOC21412156 (CHS), LOC21396296 (F3H) and LOC21388202 (F3′H) in CK were higher than those in BS-treated samples, whether in tender leaves or mature leaves. The expression levels of LOC21411675 and LOC21412648 in Treat-TL were significantly lower than that in CK-TL, but there was no significant difference between the expression levels of LOC21411675 and LOC21412648 in Treat-ML and CK-ML. These results were consistent with the transcriptome data coming from RNA-Seq analysis (Figure 10), which indicate that the transcriptome data were highly reliable. These data could serve as a valuable reference for screening key genes involved in the biosynthesis of alkaloids and flavonoids in mulberry leaves.

## 4. Discussion

### 4.1. Impact of BS on Flavonoids and Alkaloids Accumulation

Flavonoids and alkaloids are very important secondary metabolites in mulberry leaves. Among the various factors that could affect plant secondary metabolite accumulation, PGPR have gained considerable attention. In this study, application BS agents resulted in a decrease in the synthesis of flavonoids, as well as an increase in the synthesis of DNJ in mulberry leaves. This is consistent with previous studies showing that inoculation with PGPR has the potential to increase alkaloid yield in plants [35,36] but inconsistent with the results stating that *B. subtilis*-treated plants exhibited significantly improved synthesis of flavonoids [26,37,38].

The changes in flavonoids and alkaloids maybe attributed to activation of plant defense responses and regulation of plant hormone levels, as the DEGs in different groups were enriched via plant–pathogen interaction and plant hormone signal transduction (Figure 3B,C). The application of endophyte *B. subtilis* was known to induce systemic resistance in plants, which involves the up-regulation of defense-related genes and the production of defensive substance such as phenolic compounds and flavonoids [39,40]. Alkaloids act as mediators of stress responses, protecting plants from the detrimental effects of environmental challenges [41]. Plant hormones, including auxins, gibberellins and jasmonates, play important roles in regulating alkaloid production. *B. subtilis* has been reported to influence the levels of various plant hormones that participate in the regulation of secondary metabolism [40,42].

Leaf maturity also significantly influences the accumulation of flavonoids and alkaloids in plants. We found that the DNJ contents in tender leaves were significantly higher than those in mature leaves, in accordance with previous studies [13,14]. The young leaves have been reported to exhibit high enzymatic activity, thereby enhancing the biosynthesis of DNJ [13,14]. Notably, the DNJ contents in tender leaves increased significantly, while those in mature leaves did not show significant changes after the application of *B. subtilis* in this study, indicating that young leaves maintain a different balance of defense responses compared to mature leaves [43]. Furthermore, leaf age can influence hormone cross talk [44].

### 4.2. Gene Expression Associated with Flavonoids and Alkaloids Synthesis

The flavonoid biosynthesis pathway initiates with the condensation of p-coumaroyl-CoA, catalyzed by various kinds of enzymes (Figure S2). Quercetin glycosides and kaempferol glycosides were the predominant flavonoids in mulberry leaves [45]. In the present investigation, the flavonoid content was decreased in BS-treated mulberry leaves. Correspondingly, most genes including CHS, F3′H, F3H, HCT and UGT were down-regulated in BS-treated samples (Figure 8). CHS, the key enzyme that catalyzes the condensation of p-coumaroyl-CoA, resulting in the formation of naringenin chalcone, directs the phenylpropanoid metabolic pathway to the flavonoid biosynthesis pathway [46,47]. Hydroxylases (F3H and F3′H) introduce hydroxyl groups into the flavonoid backbone, leading to the formation of kaempferol and quercetin [33,48]. Many researchers have found that increased expression of CHS, F3H or F3′H could enhance the production of flavonoids [49,50]. HCT catalyzes the synthesis of p-coumaroyl-CoA to generate p-coumaroyl-shikimic acid or p-coumaroyl-quinic acid, which is then hydroxylated to generate caffeoyl shikimic acid or caffeoyl quinic acid (Figure 8) [51,52]. 5-O-caffeoylshikimic acid and 5-O-p-coumaroylquinic acid were positively correlated with HCT expression levels, in accordance with the reports that HCT promotes caffeoyl quinic acid synthesis [53,54]. Therefore, we believe that the decrease in flavonoid content was mainly due to the change in the transcription and expression level of the CHS, F3H, F3′H, FLS and HCT genes.

In this study, as the most important piperidine alkaloid in mulberry leaves, DNJ was found to accumulate in tender leaves, especially in BS-treated tender mulberry leaves. Meanwhile, the contents of piperidine, as well as the transcription levels of one AOC gene, were higher in BS-treated samples (Tables S3 and S9). This corresponds to a previous report stating that DNJ is derived from lysine and catalyzed by AOC and lysine decarboxylase (LDC) [55]. However, there were only six DEGs encoding three enzymes (AK, LL-DAP-AT, AOC) associated with the DNJ synthesis pathway after BS treatment (Figure 9, Table S9). This suggests that part of the genes affecting DNJ biosynthesis might not be represented in the current lists of DEGs or that the thresholds for significance and fold change were set too high for DEGs [34]. Yang et al. [56] showed that DNJ was derived from sugar in mulberry, but the intermediate 2-amino-2-deoxy-D-mannitol (ADM) and ADM dehydrogenase were not yet detected in our study.

The biosynthesis of piperidine alkaloids was crossed with tropane alkaloids biosynthesis pathway, as well as L-phenylalanine metabolism (Figure S3). The conversion of precursors into alkaloids involves several key reactions, which are catalyzed by AOC, CHS, tropinone reductase, aspartate aminotransferase, tyrosine aminotransferase, etc. (Figure S3). It is noteworthy that CHS expression levels were almost positively correlated with flavonoid content but negatively correlated with L-phenylalanine or DNJ (Table S8), indicating that CHS not only directly influences the production of flavonoids but also regulates the synthesis of alkaloids. Moreover, the content of L-phenylalanine increased significantly in BS-treated leaves, but the content of corresponding alkaloids had no significant change (Figure S3). The alkaloid biosynthesis pathway includes many branches; therefore, the regulatory mechanisms governing the related enzymes within the metabolic network need further exploration.

## 5. Conclusions

Through metabolomics and transcriptome analysis, we obtained 904 DSMs and 9715 DEGs in mulberry leaves after *B. subtilis* application, indicating that *B. subtilis* exerted a profound influence on the metabolic profiling and gene expression levels of mulberry leaves. Specifically, the application of *B. subtilis* significantly inhibits the production of flavonoids in both tender and mature leaves, while it promoted the accumulation of DNJ in tender leaves. The mechanisms underlying this influence might involve the activation of plant defense responses, modulation of plant hormone levels, and regulation of the expression of related genes. CHS, CHI, F3H, F3′H, FLS and HCT exhibited a strong correlation with flavonoid synthesis. AOC, AK and LL-DAP-AT were highly related to the DNJ biosynthesis. These findings highlight the potential of *B. subtilis* for promoting the production of valuable secondary metabolites. It should be noted that *B. subtilis* may inhibit the production of other components while promoting the synthesis of a specific active ingredient active component. This study also contributed to our understanding of the molecular mechanisms underlying the accumulation of flavonoids and alkaloids in mulberry leaves. Considering that the biosynthesis of alkaloids and flavonoids involves complex pathways, further research is imperative to gain a comprehensive understanding of the precise functional roles of specific genes. It is also necessary to investigate the manner in which plant hormones regulate the biosynthesis of alkaloids and flavonoids.

## Data Availability

Raw reads have been deposited as a NCBI SRA BioProject under accessions PRJNA1060472 (SUB14115057). The original contributions presented in this study are included in the article and Supplementary Materials.

## Supplementary Materials

The following supporting information can be downloaded via this link: https://www.mdpi.com/article/10.3390/metabo14040180/s1, Figure S1: KEGG enrichment of the differentially synthesized metabolites and genes; Figure S2: Flavonoid synthesis pathway map; Figure S3: Tropane, piperidine and pyridine alkaloids biosynthesis pathway map; Table S1: The primers used for qRT-PCR; Table S2: The metabolites of all samples; Table S3: Summary of the significantly different metabolites; Table S4: All the functionally annotated genes; Table S5: All the differentially expressed genes; Table S6: Metabolite loading results of O2PLS analysis; Table S7: Gene loading results of O2PLS analysis; Table S8: The correlation between the differentially synthesized metabolites and the differentially expressed genes involved in flavonoid and alkaloids biosynthesis pathway; Table S9: The expression levels of the related genes involved in flavonoid and 1-deoxynojirimycin biosynthesis in mulberry leaves.
