# Supplementary material for: Soil Application of Bacillus subtilis Regulates Flavonoid and Alkaloids Biosynthesis in Mulberry Leaves
Source: Metabolites. 2024 Mar 23;14(4):180. doi: 10.3390/metabo14040180 (PMC11052171; doi:10.3390/metabo14040180)
Supplement: Supplementary file 1 [file metabolites-14-00180-s001.zip › Table S1-Primers.pdf]

Table 1 Real-time PCR primer sequence and reaction procedure

Target gene	Prime	Primer sequences (5'-3')
β-Actin	β-Actin-F	CATTGTCTTGTCTGGTGGTT
	β-Actin-R	TCATCATACTCCGACTTTGC
LOC21395595	LOC21395595-F	CAACGACGATTACCCGCAAACTC
	LOC21395595-R	CCTGCCCACTTCTCCGACTTG
LOC21406007	LOC21406007-F	TCACTGTGGATTTGGGCTTGGG
	LOC21406007-R	ACCTCTTGCGGCGATAGATTCAG
LOC21411755	LOC21411755-F	GCCACTGTGCTCTCCATCGG
	LOC21411755-R	TGTTGGTGATGCGGAAGTAGTAGTC
LOC21412156	LOC21412156-F	CGCAGACGATCCTTCCAGACTC
	LOC21412156-R	GAATGCCTCCACCAGACTCTTCTC
LOC21411675	LOC21411675-F	TTTGCTCCGAACTCACCATCCC
	LOC21411675-R	AGACCCACCATCAGCGAAGATTG
LOC21412648	LOC21412648-F	CTCCGAACTCACCATTCCAACTTTC
	LOC21412648-R	ATCACAGCCGACGCACCATC
LOC21396296	LOC21396296-F	GCCTCCCTCTCGCATCAACTAC
	LOC21396296-R	GAACACCTTATCCGCCACCTCTC
LOC21388202	LOC21388202-F	GGATTTGTGCGGGTATGAGTTTGG
	LOC21388202-R	TTTCAGGTAGCAGCCCATTAGGTAG
LOC21393738	LOC21393738-F	TCGGCGTGTTCTTCTGCTATGG
	LOC21393738-R	AAAGGCGTAGAAGGACACCAAGG
LOC21405124	LOC21405124-F	ACGTCCATCCAAGTGAAGTTCTCC
	LOC21405124-R	CTCGTTCATCCGTTATCGCCTTTG
LOC21404779	LOC21404779-F	GAAGGTTGAGACTGTTTGCGTGAC
	LOC21404779-R	ACGGTTGCTCGGACTGTGTAATC
LOC21392651	LOC21392651-F	CATTCCCGCCAATTTACCCTGTG
	LOC21392651-R	CCGAGGAAGTAGGCTGTCTATCAAG
