# Supplementary figures and images for: Soil Application of Bacillus subtilis Regulates Flavonoid and Alkaloids Biosynthesis in Mulberry Leaves
Source: Metabolites. 2024 Mar 23;14(4):180. doi: 10.3390/metabo14040180 (PMC11052171; doi:10.3390/metabo14040180)

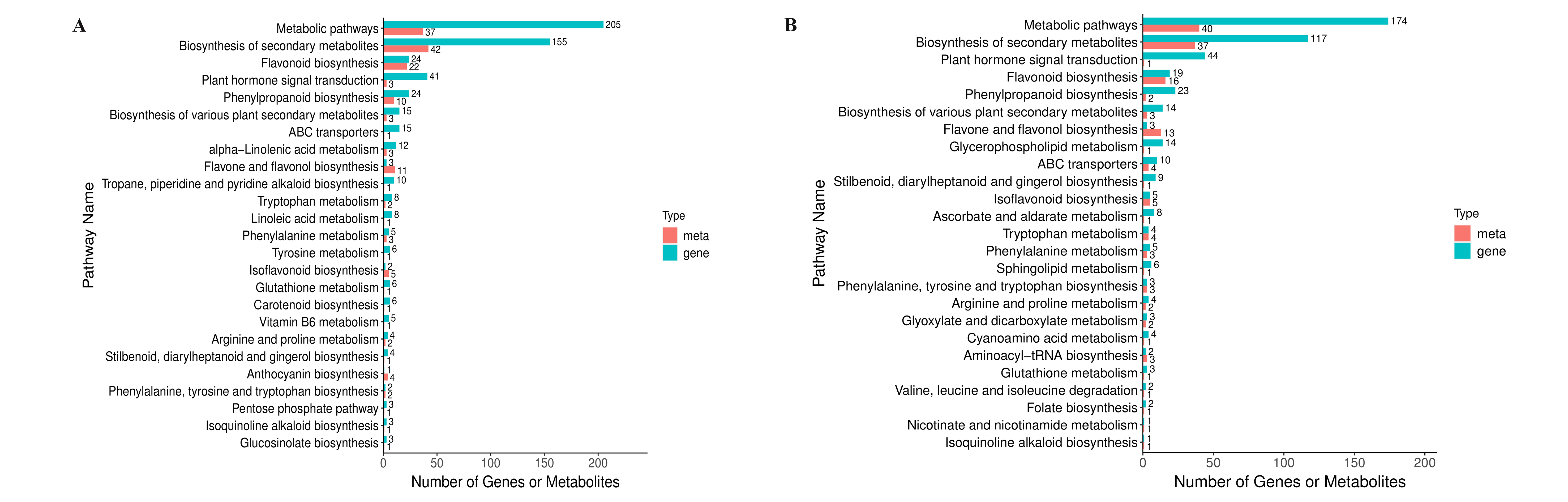

Supplement: Supplementary file 1 [file metabolites-14-00180-s001.zip › Figure S1.jpg]

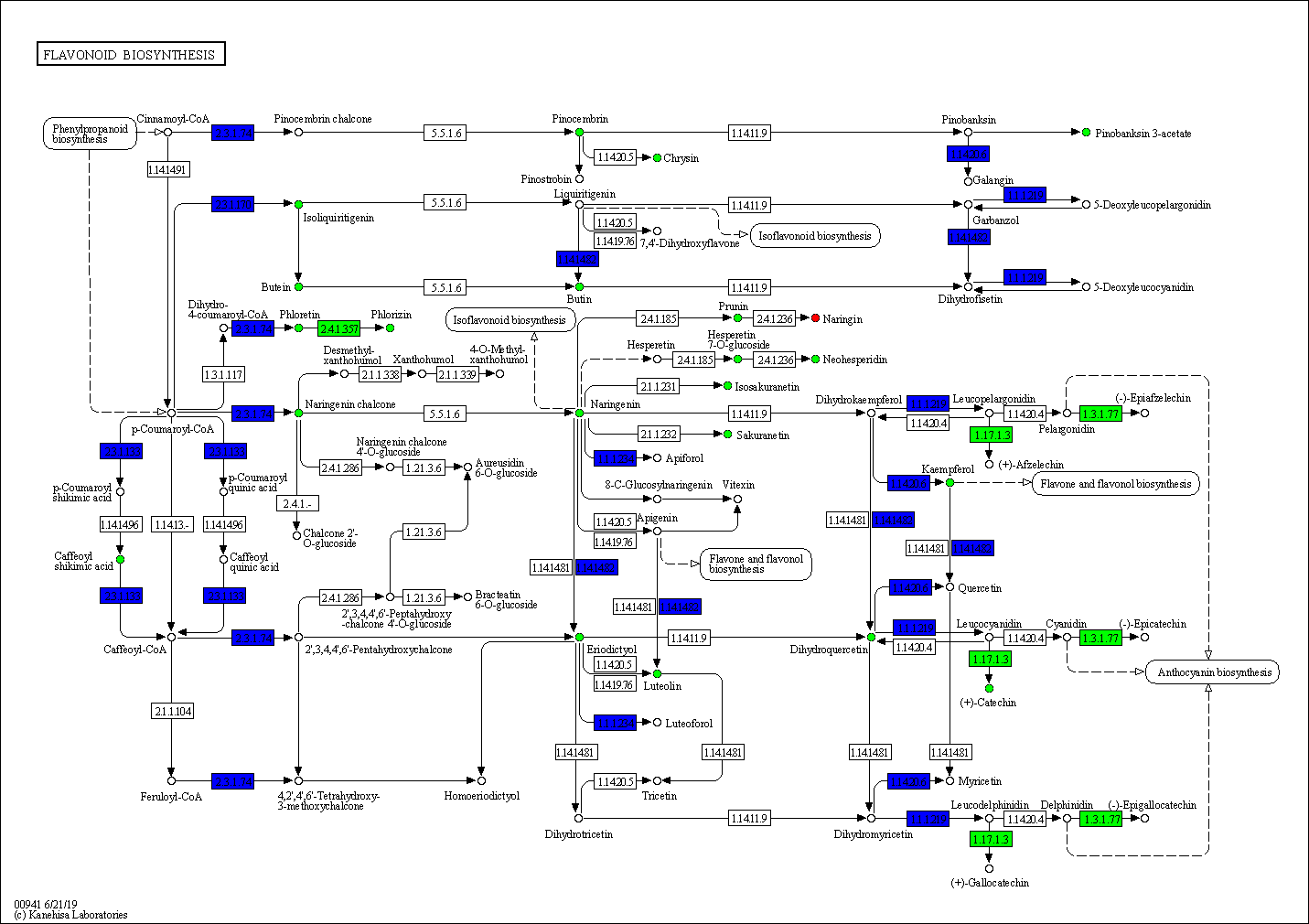

Supplement: Supplementary file 1 [file metabolites-14-00180-s001.zip › Figure S2-ko00941.png]

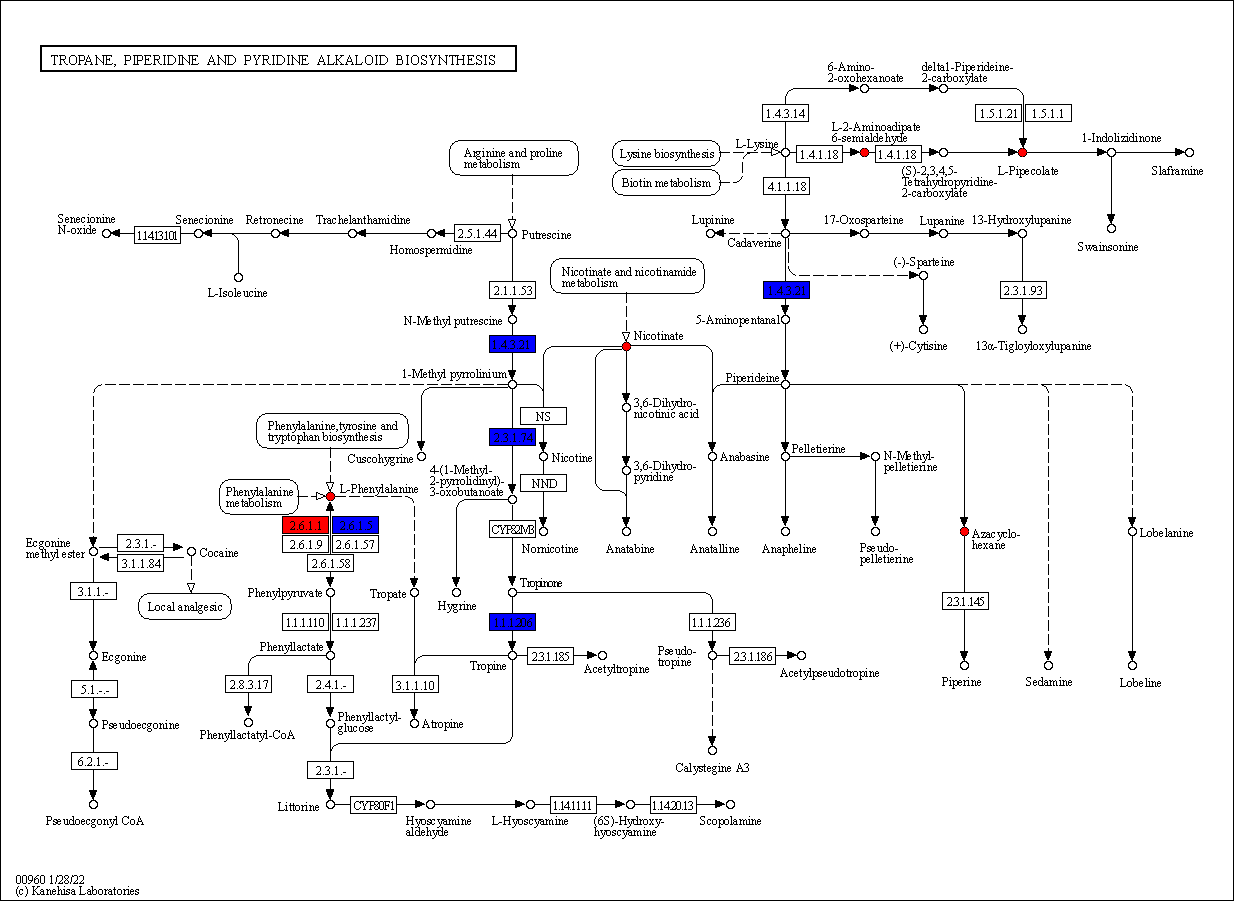

Supplement: Supplementary file 1 [file metabolites-14-00180-s001.zip › Figure S3-ko00960.png]
